# Prophage Rs551 and Its Repressor Gene *orf14* Reduce Virulence and Increase Competitive Fitness of Its *Ralstonia solanacearum* Carrier Strain UW551

**DOI:** 10.3389/fmicb.2017.02480

**Published:** 2017-12-22

**Authors:** Abdelmonim Ali Ahmad, Michael J. Stulberg, Qi Huang

**Affiliations:** ^1^Floral and Nursery Plants Research Unit, United States National Arboretum, United States Department of Agriculture–Agricultural Research Service, Beltsville, MD, United States; ^2^Department of Plant Pathology, Faculty of Agriculture, Minia University, El-minia, Egypt

**Keywords:** *Ralstonia solanacearum*, filamentous lysogenic phage, prophage, phage repressor, competitive fitness, race 3 biovar 2, phylotype, sequevar

## Abstract

We previously characterized a filamentous lysogenic bacteriophage, ϕRs551, isolated directly from the race 3 biovar 2 phylotype IIB sequevar 1 strain UW551 of *Ralstonia solanacearum* grown under normal culture conditions. The genome of ϕRs551 was identified with 100% identity in the deposited genomes of 11 race 3 biovar 2 phylotype IIB sequevar 1 strains of *R. solanacearum*, indicating evolutionary and biological importance, and ORF14 of ϕRs551 was annotated as a putative type-2 repressor. In this study, we determined the effect of the prophage and its ORF14 on the virulence and competitive fitness of its carrier strain UW551 by deleting the *orf14* gene only (the UW551 *orf14* mutant), and nine of the prophage’s 14 genes including *orf14* and six out of seven structural genes (the UW551 prophage mutant), respectively, from the genome of UW551. The two mutants were increased in extracellular polysaccharide production, twitching motility, expression of targeted virulence and virulence regulatory genes (*pilT, egl, pehC, hrPB, and phcA*), and virulence, suggesting that the virulence of UW551 was negatively regulated by ϕRs551, at least partially through ORF14. Interestingly, we found that the wt ϕRs551-carrying strain UW551 of *R. solanacearum* significantly outcompeted the wt strain RUN302 which lacks the prophage in tomato plants co-inoculated with the two strains. When each of the two mutant strains was co-inoculated with RUN302, however, the mutants were significantly out-competed by RUN302 for the same colonization site. Our results suggest that ecologically, ϕRs551 may play an important role by regulating the virulence of and offering a competitive fitness advantage to its carrier bacterial strain for persistence of the bacterium in the environment, which in turn prolongs the symbiotic relationship between the phage ϕRs551 and the *R. solanacearum* strain UW551. Our study is the first toward a better understanding of the co-existence between a lysogenic phage and its carrier plant pathogenic bacterial strain by determining the effect of the prophage Rs551 and its repressor on the virulence and competitive fitness of its carrier strain UW551 of *R. solanacearum*.

## Introduction

Bacterial wilt, a soil borne vascular disease caused by *Ralstonia solanacearum*, is one of the most devastating bacterial diseases in tropical, subtropical, and temperate regions of the world. The bacterium has a host range of over 450 plant species, including ornamentals such as geranium and economically important crops such as tomatoes and potatoes (Hayward, 1991; Kelman et al., 1994; Denny, 2006). *R*. *solanacearum* normally invades host plants from soil through wounds in roots, reproduces in the xylem vessels, and spreads rapidly through the plant’s vascular system resulting in wilting and death of the plant (Vasse et al., 2000). The bacterium can be spread in soil or water or through latently infected plant materials like potato tubers or geranium cuttings.

*Ralstonia solanacearum* is historically classified into five races and five biovars based on host range and biochemical properties, respectively. Molecular classification, however, has thus far grouped *R. solanacearum* into 4 phylotypes and 53 sequevars (Albuquerque et al., 2014; Stulberg and Huang, 2016). The race 3 biovar 2 (phylotype IIB sequevars 1 and 2) of *R. solanacearum* causes devastating brown rot of potato, and is a quarantine pathogen in many countries and listed as a select agent in the United States (Agricultural Bioterrorism Protection Act of 2002, 2002).

Biochemical and molecular genetic studies over the past 60 years have explored the underlying mechanisms of *R. solanacearum* pathogenesis. Several major virulence factors have been identified, including extracellular polysaccharides (EPSs), plant cell wall degrading enzymes such as the β-1,4-endoglucanase (Egl) (Roberts et al., 1988), endopolygalacturonase (PehA) (Schell et al., 1988; Allen et al., 1991), and exopolygalacturonases (PehB and PehC) (Huang and Allen, 2000; González and Allen, 2003), as well as type IV pili (Tfp) that is essential for twitching motility, adherence and colonization, and biofilm formation (Liu et al., 2001; Kang et al., 2002). The expression of these virulence genes is controlled by PhcA, a LysR-type global transcriptional regulator (Schell, 2000). *R. solanacearum* also uses a key transcriptional activator, HrpB, to drive the expression of *hrp* genes encoding a type III secretion system and effector molecules that allow translocation of the effector proteins into plant cells for pathogenicity (Genin and Boucher, 2004; Delaspre et al., 2007).

Control of bacterial wilt is mainly achieved by exclusion and eradication. For the past 10 years, a wide range of lysogenic and lytic bacteriophages specifically infecting *R. solanacearum* have been isolated from soil of crop fields, and their use as potential biocontrol agents has been explored (Yamada et al., 2007; Fujiwara et al., 2011; Bhunchoth et al., 2015). We recently isolated a filamentous lysogenic bacteriophage ϕRs551 directly from the race 3 biovar 2 strain UW551 of *R. solanacearum* grown under normal culture conditions (Ahmad et al., 2017). The phage has a particle size of about 1,200 nm in length and 7 nm in width, and has a genome size of 7,929 nucleotide with 14 open reading frames (Ahmad et al., 2017). In contrast with other *R. solanacearum* phages isolated from soil, ϕRs551 is the first isolated phage that contains a resolvase (ORF13) and a putative type-2 phage repressor (ORF14), although how this repressor maintains a prophage state and phage immunity in ϕRs551 had not been studied. In addition, the genome sequence of ϕRs551 is surprisingly found with 100% identity in the deposited genomes of 11 race 3 biovar 2 phylotype IIB sequevar 1 strains of *R. solanacearum*, indicating evolutionary importance (Ahmad et al., 2017). Infection of a susceptible *R. solanacearum* strain RUN302 by ϕRs551 resulted in colonies with less fluidal appearance and reduced EPS production, motility, and virulence (Ahmad et al., 2017). It is unclear, however, what effects the phage or the putative phage repressor has on its host strain *R. solanacearum* UW551.

Huerta et al. (2015) recently hypothesized that differences in temperature adaptation and competitive fitness account for the uneven geographic distribution of *R. solanacearum* strains, and found that lowland tropical and warm temperate strains out-compete temperate strains of *R. solanacearum*, probably due to bacteriocins produced by the tropical and warm temperate strains to specifically inhibit the growth of the temperate strains. *R. solanacearum* lytic phages encode bacteriolytic proteins (Ozawa et al., 2001) and are lytic to susceptible strains (Fujiwara et al., 2011), and lysogenic phages are known to bring novel phenotypic properties that might affect the fitness of their host bacteria (Casjens, 2003; Brüssow et al., 2004; Canchaya et al., 2004; Davies et al., 2016). We therefore hypothesize that *R. solanacearum* strains prevalent under different environmental conditions may contain different phages that offer competitive fitness to their host strain, allowing the host strains to persist in the environment by preventing the establishment of susceptible strains of *R. solanacearum* that lack the lysogen.

To better understand the contribution of the putative type-2 repressor of ϕRs551 and the phage ϕRs551 itself to the virulence and competitive fitness of its carrier strain UW551 of *R. solanacearum*, we generated two *R. solanacearum* mutants by knocking out the *orf14* gene, and nine of the 14 genes of ϕRs551 from the prophage region of UW551, respectively. The phenotype of the two mutants included increased EPS production and twitching motility, increased expression of five other genes tested, as well as increased virulence when inoculated into tomato plants alone. When co-inoculated with strain RUN302 which lacks the prophage Rs551 in its genome for infection of tomato plants, these mutants also had decreased competitive fitness in colonizing tomato stems and had little effect on the virulence of strain RUN302.

## Materials and Methods

### Bacterial Strains and Plasmids

Bacterial strains and plasmids used and constructed in this study are listed in **Table 1**.

**Table 1:** Bacterial strains and plasmids used in this study.

Designation	Relevant characteristics^a^	Source or reference
**Strains**		
*Ralstonia solanacearum*		
UW551	Wild-type, race 3 biovar 2, phylotype IIB sequevar 1, ϕRs551 carrier	C. Allen, United States
UW551ΔϕRs551-*orf14* (UW551 *orf14* mutant)	UW551 with a 342-bp prophage region containing *orf14* replaced with a 616-bp Gm cassette, Gm^R^	This study
UW551ΔϕRs551 (UW551 prophage mutant)	UW551 with a 3,321-bp prophage region including *orf14* and *orf1* to *orf8* replaced with a 616-bp Gm cassette, Gm^R^	This study
RUN302	Wild-type, biovar 1, phylotype IIB sequevar 4, ϕRs551^S^	P. Prior, France
*Escherichia coli*		
TOP10	F- *mcr*A Δ(*mrr*-*hsd*RMS-*mcr*BC) Φ80*lac*ZΔM15 Δ*lac*X74 *rec*A1 *ara*D139 Δ(*ara*-*leu*)7697 *gal*U *gal*K *rps*L (StrR) *end*A1 *nup*G	Invitrogen
TP997	MG1655 *lacIP*Δ*::bla-aadA1148 galK::aacC1067*	Addgene
**Plasmid**		
pCR^TM^ Blunt II TOPO	PCR cloning vector, Kan^R^ Zc^R^	Invitrogen
pCR Blunt II-Gm	pCR^TM^ Blunt II TOPO with a 616-bp Gm cassette, Gm^R^ Kan^R^ Zc^R^	This study
pCR Blunt II-ϕRs551-*orf14* Up-Gm-ϕRs551-*orf14* Down	pCR Blunt II-Gm with 994-bp upstream and 819-bp downstream fragments of the 342-bp prophage region in *R. solanacearum* inserted before and after the Gm cassette, respectively, Gm^R^ Kan^R^ Zc^R^	This study
pCR Blunt II-ϕRs551 Up-Gm-ϕRs551 Down	pCR Blunt II-Gm with 994-bp upstream and 850-bp downstream fragments of the 3,321-bp prophage region in *R. solanacearum* inserted before and after the Gm cassette, respectively, Gm^R^ Kan^R^ Zc^R^	This study

*^a^Gm^R^, Kan^R^, and Zc^R^ indicate resistance to gentamicin, kanamycin, and zeocin, respectively.*

### Growth and Isolation of Bacterial Strains

*Ralstonia solanacearum* was grown and its inocula prepared as described (Stulberg et al., 2015). To isolate *R. solanacearum* from inoculated plant samples, 0.5-cm plant stem sections were prepared and homogenized as described (Stulberg and Huang, 2015), and dilution plated onto modified semi-selective medium agar plates (Huang and Lakshman, 2010). *Escherichia coli* strains were cultured at 37°C in Luria-Bertani medium (Miller, 1972). When needed, antibiotics were added at 25 μg/ml for kanamycin and 15 μg/ml for gentamicin. Since *R. solanacearum* strain UW551 is a select agent pathogen in the United States, manipulation of the strain was conducted in a secured laboratory and virulence assays described below were performed in a secured greenhouse section approved for select agent research by USDA/APHIS using standard operating procedures also approved by APHIS for race 3 biovar 2 strains of *R. solanacearum*.

### DNA Isolation and Manipulation

Standard molecular biology techniques were used for plasmid isolation, restriction digestion, cloning, and transformation of *E. coli* strains (Sambrook and Russell, 2001). Total bacterial DNA was extracted using Qiagen’s Blood and Tissue Kit (Qiagen, Chatsworth, CA, United States) following the manufacturer’s instructions.

### Design of PCR Primers and PCR Conditions

Primers designed in this study were listed in **Table 2**. They were designed based on the deposited UW551 draft genome sequence in GenBank (ASM16795v1, GCA_000167955). The regions selected for primer design were entered into the free online A plasmid Editor (ApE) program. Similar design parameters (GC = 45–60%, Tm = 60–64°C, primer length 18–26) were used for primers in each pair. The specificity of each primer pair and amplicon was checked by BLASTn against the UW551 genome, and the nr and WGS databases in GenBank for specificity.

**Table 2:** List of primers designed in this study.

Primer pair	Sequence (5′-3′, restriction enzyme sites are underlined)	Size of PCR product (bp)
ϕRs551-*orf14-*up-F-XbaI	TATAATCTAGAGGATATGGAGGTGGCGCATG	994
ϕRs551-*orf14*-up-R-XhoI	AGTATCTCGAGCTAAGCACGGGAGGAGTTCG	
ϕRs551-down-F-BamHI	TTACTGGATCCACGAACACGACAACCAACAC	850
ϕRs551-down-R-KpnI	AATCTGGTACCAAAGCGTCACGACCTTGC	
ϕRs551-*orf14*-down-F-BamHI	ATACTGGATCCCTGCATGTCACTCCGAACG	819
ϕRs551-*orf14-*down-R-KpnI	ACAAAGGTACCAACTCTTCCAGACAGCCCAC	
*Egl-*F	TCATCAGCCCGAAGATGAC	140
*Egl*-R	GCTCGATCCGCACAACTAT	
*pilT*-F	GTAATGCTTGCGCTGCAC	147
*pilT*-R	GCGTCTGATCTGCACTTGTC	
*pehC*-F	GTTGTTCGGATTGCTGTACG	227
*pehC*-R	AGTCAAACGATTGCCTGAACTA	
*hrpB*-F	TTCTCGATGATGTAGCGATAGG	123
*hrpB*-R	CACCGAGACGGTCAACCT	
*phcA*-F	GTGTATTCGGCCACCACCT	147
*phcA*-R	CGAGGCCTACAGCCTCAAC	

Colony PCR was performed by picking *R. solanacearum* cells using a sterile toothpick or pipette tip from a single colony grown on a plate and mixing the cells in 100 μl of sterile water. The cell suspension was boiled for 5 min and cooled on ice or stored at -20°C until use. Two to five microliters of the suspension were used for PCR.

PCR to amplify the upstream and downstream prophage regions was conducted in a 20 μl volume containing 1x KAPA HiFi HotStart ReadyMix (Kapa Biosystems, Boston, MA, United States), 5 pmol of each primer, and approximately 20 ng of DNA template. PCR conditions were 1 cycle of 3 min at 95°C, followed by 30 cycles of 20 s at 98°C, 15 s at 58°C, and 30 s at 72°C, with a final extension of 2 min at 72°C. PCR to amplify virulence-related genes was conducted in a 20-μl volume containing 1x GoTaq Green Master Mix (Promega, Madison, WI, United States), 20 ng of template DNA, and 5 pmol of each primer. PCR conditions were 1 cycle of 4 min at 94°C, 30 cycles of 1 min at 94°C, 1 min at 60°C, and 1 min at 72°C, with a final extension of 10 min at 72°C.

### Construction of *R. solanacearum* Mutants

**FIGURE 1:**
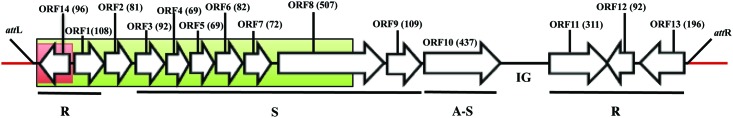
Genomic organization of ϕRs551’s identical prophage region in *R. solanacearum* strain UW551. Bacterial sequence is in red and the prophage sequence in black. The regions in red and green boxes were replaced with a 616-bb gentamicin cassette in UW551’s *orf14* mutant and prophage mutant strains, respectively. The open reading frames (ORF) in the prophage are represented by arrows with indicated direction of transcription, and the number of amino acids in each ORF is indicated. R, S, and A-S represent functional modules for replication, structure, and assembly and secretion, respectively. IG represents intergenic region. *att*L and *att*R indicate the location of ϕRs551’s left and right attachment sites, respectively.

To study the role of *orf14* of the prophage Rs551 and the prophage itself in *R. solanacearum*, two mutants of *R. solanacearum* which had deletions in the prophage were constructed in strain UW551 by homologous double recombination. Mutant UW551ΔϕRs551-*orf14*, designated as the UW551 *orf14* mutant, was generated by replacing a 342-bp prophage region, located in contig 0570 of UW551 (GenBank accession number: AAKL01000012.1), with a 616-bp gentamicin cassette. The 342-bp fragment contained the entire 291-bp prophage region corresponding to ϕRs551’s *orf14*, coding for a putative type-2 phage repressor, as well as 42-bp upstream and 9-bp downstream of *orf14*. Mutant UW551ΔϕRs551, designated as the UW551 prophage mutant, was constructed by replacing a 3,321-bp prophage region with a 616-bp gentamicin cassette. The 3,321-bp fragment contained the same 342-bp in the *orf14* mutant, as well as an additional 2,979-bp located in the same contig of UW551, corresponding to ϕRs551’s *orf1*in the replication module, *orf2* between the replication and structure modules, and six (*orf3* to *orf7*, and 1,005-bp of the 1,524-bp of *orf8*) of the seven structural genes in the structural module of the prophage (**Figure 1**). To make the mutants, a 616-bp gentamicin cassette was first amplified from a colony of TP997, purchased from Addgene (Cambridge, MA, United States), by PCR with primers 5′-CGAATCCATGTGGGAGTTTA-3′ and 5′-TTAGGTGGCGGTACTTGGGT-3′ (Poteete et al., 2006). The cassette was then cloned into the TOPO site of the vector pCR Blunt II TOPO to generate pCR Blunt II-Gm using Invitrogen’s Zero Blunt^®^ TOPO^®^ PCR Cloning Kit according to the manufacturer’s instructions. Two regions of DNA, 994-bp in size located upstream and 815-bp downstream of the 342-bp prophage region, were amplified by PCR using primers in **Table 2**, digested with respective restriction enzymes, and cloned sequentially into the multiple cloning sites before and after the gentamicin cassette in pCR Blunt II-Gm to obtain pCR Blunt II-ϕRs551-*orf14* Up-Gm-ϕRs551-*orf14* Down. Similarly, the same 994-bp upstream fragment and a 850-bp downstream fragment of the 3,321-bp prophage region was cloned sequentially to obtain pCR Blunt II-ϕRs551 Up-Gm-ϕRs551 Down. The resulting plasmids were electroporated into competent cells of *R. solanacearum* strain UW551 as described by Ahmad et al. (2017). This was followed by selection on gentamicin-containing triphenyltetrazolium chloride (TZC) plates (Kelman, 1954) for transformants that had undergone homologous double recombination between the Up-Gm-Down region in pCR Blunt II-Up-Gm-Down and the Up and Down prophage sequences in the chromosome of UW551 of *R. solanacearum.* The knock-out mutants were identified by screening on TZC plates amended with gentamicin, and confirmed by PCR using primer pairs located within the sequences of the mutated regions that showed a lack of any amplified products. To determine if a phage was still produced from the UW551 mutants, an aliquot from the supernatant of the *R. solanacearum* mutants was subjected to the spot test and plaque-forming assay (Ahmad et al., 2017) using *R. solanacearum* RUN 302, a strain susceptible to ϕRs551 and which contained no ϕRs551 sequence in its genome before infection. The presence or absence of phage particles in the supernatant of the *R. solanacearum* mutants was also examined under transmission electron microscope (Ahmad et al., 2017).

### EPS, *in Vitro* Growth, and Twitching Motility Assays

Extracellular polysaccharide in the supernatant of *R. solanacearum* was determined quantitatively, *in vitro* growth of *R. solanacearum* strains were measured, and twitching motility examined as described by Ahmad et al. (2017), except that twitching motility was visualized using a Zeiss AxioZoom v16 *stereo zoom microscope* (Carl Zeiss Microscope GmbH, Germany). Two replicates were used for each strain in the EPS assay and the experiment was repeated three times.

### RNA Isolation and Analysis of Gene Expression

Total bacterial RNA was isolated from 3 ml of *R. solanacearum* culture at the exponential growth phase (OD_600_ = 0.3) using Qiagen’s RNeasy Protect Bacterial and RNeasy Mini Kits (Qiagen, Inc., CA) according to the manufacturer’s protocol. Ambion^®^ TURBO DNA-*free^TM^* DNase Treatment and Removal Reagents (Life Technology) were used to remove contaminating DNA from the RNA preparation and to subsequently remove the DNase and divalent cations from the sample. The absence of DNA contaminants was confirmed by PCR using gene-specific primers (**Table 2**) on the RNA samples. The bacterial genomic DNA of *R. solanacearum* strain UW551 was used as positive, and sterile water negative controls. The RNA samples were quantified using the Nanodrop ND-1000 spectrophotometer (NanoDrop Tech. Inc.). One microgram of the RNA was reverse transcribed using Quantabio’s qScript^TM^ cDNA SuperMix (Quantabio, Beverly, MA, United States) according to the manufacturer’s instructions. Quantitative PCR analysis was carried out with gene-specific primers (**Table 2**) using 1 μl of cDNA as a template in a 20 μl volume containing 10 μl of IQ^TM^ SYBR^®^ Green Supermix (Bio-Rad) and 0.5 μM each of the gene primers. Cycling conditions were 95°C for 3 min, followed by 45 cycles of 95°C for 10 s, 60°C for 15 s, and 72°C for 30 s. At the end of the program, melting curve (65–95°C with a heating rate of 0.5°C/min) was analyzed to confirm the specificity of the primer set (Addy et al., 2012a). Relative levels of gene expression were determined using the 2^-ΔΔC_T_^ method (Livak and Schmittgen, 2001), with the 16s rRNA gene as the internal control (Addy et al., 2012a). The experiments were performed three times with two replicates each time.

### Virulence and Competition Assays

For virulence assays, tomato plants were seeded, transplanted, inoculated by soil drenching with 50 ml of *R. solanacearum* (5 × 10^7^ cells/ml) (Ahmad et al., 2017). Inoculated plants were rated using a disease index (DI) ranging from 0 (healthy) to 4 (76–100% leaves wilted) (Roberts et al., 1988). There were 10 plants per treatment and the experiment was repeated three times. To determine if ϕRs551’s *orf14* or the phage itself offers any competitive advantage to its carrier strain UW551, a competition assay was similarly performed as described for the virulence assay, except that in addition to inoculation with the ϕRs551-susceptible strain RUN302 alone, tomato plants were also co-inoculated with the following two different *R. solanacearum* strains, respectively, in a 1:1 (25 ml:25 ml) ratio: (1) wt RUN302 (does not carry and is susceptible to ϕRs551) and the wt UW551 (ϕRs551-carrier), (2) RUN302 and the UW551 *orf14* mutant strain, and (3) RUN302 and the UW551 prophage mutant strain. Tomato plants inoculated with water were served as negative controls. As soon as the inoculated plants showed signs of wilting, bacterial colonies were isolated as described above. To estimate the ratio of and to differentiate between the two strains in the stem sections, 50 randomly picked colonies, 10 from each of five different wilted plants per treatment, were subjected to the multiplex PCR developed by Stulberg et al. (2015). The experiment was performed three times.

### Statistical Analysis

Data for EPS dry weight and virulence were analyzed by one-way ANOVA using web-based statistical software^1^. Means were compared using the Tukey’s Honest Significant Difference test provided by the software. The values in gene expression and competitive fitness were shown as the means of three experiments. Differences were considered statistically significant at *P* < 0.01.

## Results

### Confirmation of *R. solanacearum* Mutants and Determination of Phage Production by the Mutant Strains

The 7,929-nucleotide genome sequence of ϕRs551 corresponds to nucleotides 73,039–80,967 in contig 0570 of the deposited genome sequence of *R. solanacearum* strain UW551 (**Figure 1**). *R. solanacearum* mutants were confirmed by their ability to grow on TZC plates containing gentamicin, and by PCR for the absence of the 342-bp prophage region in the UW551 *orf14* mutant and the 3,321-bp region in the UW551 prophage mutant (data not shown). The UW551 *orf14* mutant was found to produce phage particles spontaneously in its supernatant at a rate similar to the wt strain UW551. This was shown when the supernatant of the overnight culture of the UW551 *orf14* mutant strain was subjected to the spot test and plaque-forming assay using ϕRs551-suceptable strain *R. solanacearum* RUN302, similar plaque formation was observed, and a similar number of plaques was obtained as with the supernatant of the wt UW551 (data not shown). On the contrary, no plaques were formed when the supernatant of the prophage mutant strain was subjected to the same phage susceptibility assay, and no phage particles were observed under transmission electron microscope.

### Physiological Changes Were Detected in *R. solanacearum* Mutants

**FIGURE 2:**
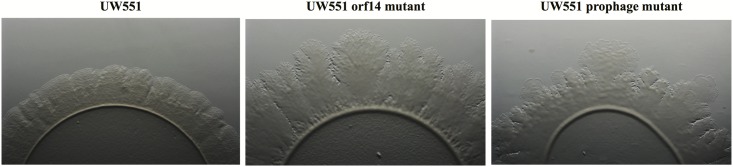
Comparison of twitching motility of *R. solanacearum* strains UW551 (left), UW551 *orf14* mutant (middle), and UW551 prophage mutant (right). Three microliters of bacterial suspension (10^8^ cells/ml) were placed in the center on a minimal medium plate, and kept for 5 days at 28°C (Ahmad et al., 2017). Visualization of twitching motility was done by placing the plate without its lid on the stage of a Zeiss AxioZoom v16 *stereo zoom microscope* (Carl Zeiss Microscope GmbH, Germany) (Ahmad et al., 2017). Corrugated trajectories formed around colonies indicate twitching motility. Note that the edge of the corrugated trajectories was smooth in UW551, but not in the two mutant strains of *R. solanacearum*.

To characterize the UW551 *orf14* and prophage mutant strains, we first compared the *in vitro* growth of the mutants with their wt strain UW551, and found all three strains grew at a similar rate (data not shown). When the three strains grew on regular TZC medium plates, however, the colonies of the mutant strains appeared more fluidal and irregular than those of the wt UW551, suggesting a high production of EPS. This observation was confirmed by an EPS quantitative assay that showed both mutant strains produced significantly higher amounts of EPS (73.6 ± 4.5 mg/10 ml for UW551 prophage mutant, and 59.6 ± 9.5 mg/10 ml for UW551 *orf14* mutant) than the wt strain (44.3 ± 7.2 mg/10 ml). The difference in EPS production between the two mutant strains was not significant. The two mutant strains also displayed distinctly different twitching motility when compared with the wt strain UW551 (**Figure 2**). For the wt strain, we observed twitching motility under a microscope as indicated by the formation of corrugated trajectories with smooth edge around the margin of its colonies (**Figure 2**, left). The size of the trajectories, however, was larger with irregular edges in *R. solanacearum* mutant strains, especially in the *orf14* mutant (**Figure 2**, middle).

### The UW551 *orf14* and Prophage Mutant Strains of *R. solanacearum* Were More Virulent than the wt Strain UW551

**FIGURE 3:**
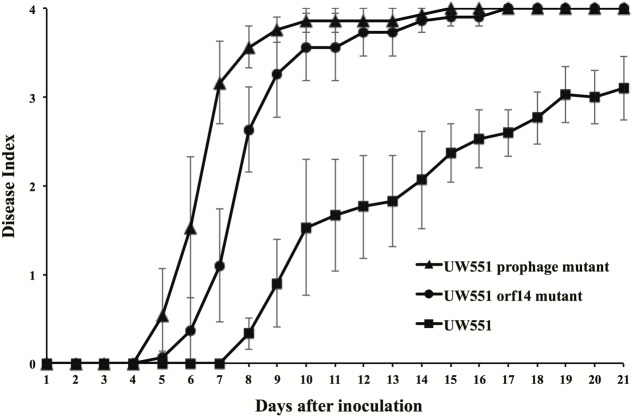
Virulence of *R. solanacearum* strains. Two- to three-week-old tomato plants were inoculated by soil drenching with 50 ml (5 × 10^7^ cells/ml) of wt UW551 (squares), UW551 *orf14* mutant (circles) or UW551 prophage mutant (triangles). Plants were rated using a DI of 0 (no wilting) to 4 (76–100% leaves wilted). Points represent means of three separate experiments with 10 plants in each experiment for a total of 30 plants. Bars indicate standard errors.

To study the effect of deletion of the targeted prophage regions in the virulence of *R. solanacearum*, we compared the virulence of the wt to that of the mutant strains of *R. solanacearum* (**Figure 3**). The wt strain UW551 did not cause any disease symptoms until 8 days after soil drenching inoculation (DI > 0), and reached a DI of 3.1 at day 21 (**Figure 3**). The virulence level caused by the two mutant strains of *R. solanacearum*, however, was significantly higher (**Figure 3**). The mutants started to cause disease symptoms 5 days after inoculation and completely wilted all inoculated plants (DI = 4) by day 17 (**Figure 3**). DIs caused by the UW551 prophage mutant were statistically similar to the ones caused by the UW551 *orf14* mutant, except at days 7 and 8 (**Figure 3**).

### Gene Expression Levels Were Increased in *R. solanacearum* Mutant Strains

**FIGURE 4:**
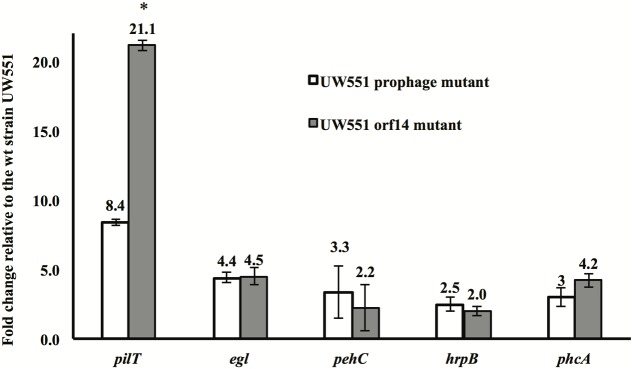
Expression of the *pilT*, *egl*, *pehC*, *hrpB*, and *phcA* genes in *R. solanacearum* UW551 *orf14* and prophage mutants, as compared to the wt strain UW551. Quantitative reverse transcription PCR from RNA extracted from *R. solanacearum* strains was performed to determine transcript levels of each gene. Each gene expression was calculated using the 2^-ΔΔC_T_^ method (Livak and Schmittgen, 2001) and normalized using the 16s rRNA gene as an internal control. Values shown are means of three separate experiments, each containing two replicates. Bars indicate standard errors. ^∗^indicates significant difference (*p* < 0.05) between the two mutants by the Student’s *t*-test.

To identify the factors contributing to increased virulence of the *R. solanacearum* mutant strains, expression of five genes (*pilT*, *egl*, *pehC*, *hrpB*, and *phcA*), all known virulence factors of *R. solanacearum*, was compared between the mutant and the wt strains. The expression of all five genes was increased in the two mutant strains, with the *pilT* gene showing the greatest increase: 8-fold in the *orf14* mutant and 21-fold in the prophage mutant (**Figure 4**). The level of expression of the *egl*, *pehC*, *hrpB*, and *phcA* genes was increased between 2.0- and 4.5-fold in the two mutants (**Figure 4**).

### *R. solanacearum orf14* and Prophage Mutant Strains of UW551 Are Out Competed by the Phage ϕRs551-Susceptible Strain RUN302 in Plants

**FIGURE 5:**
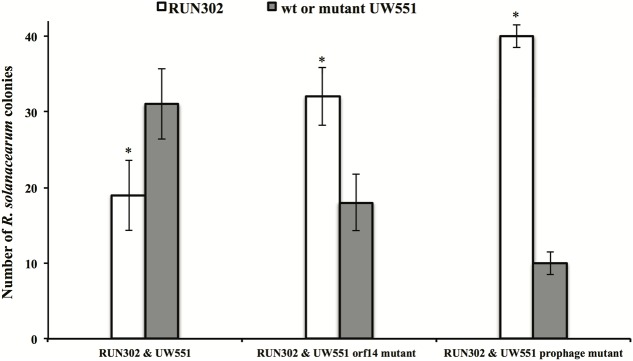
Competitive fitness of *R. solanacearum* strains in tomato stems. Two- to three-week-old tomato plants were inoculated by soil drenching with a mixture of two different *R. solanacearum* strains as indicated (25 ml:25 ml, 5 × 10^7^ cells/ml). At first sign of wilt symptoms, the population ratio of each strain was determined by serial dilution plating of ground 0.5-cm stem, followed by a multiplex PCR (Stulberg et al., 2015) to differentiate the strains. Values are means of three experiments, each containing 50 randomly picked colonies from five wilted plants per treatment. Bars indicate standard errors. ^∗^indicates significant difference (*p* < 0.05) between RUN302 and the wt or mutant UW551 strains by the Student’s *t*-test.

The effect of UW551 and its mutant strains on plant colonization by RUN302, a strain of *R. solanacearum* lacking the prophage Rs551, was studied. RUN302 was co-inoculated with one of the UW551 strains for infection of tomato plants. The ratio of the two mixed strains was determined in stem sections of infected tomato plants. When the wt ϕRs551-carrier strain UW551 was co-inoculated with the wt ϕRs551-lacking strain RNN302, 30 out of 50 randomly picked bacterial colonies isolated from the tomato stems belonged to UW551 (**Figure 5**), significantly more than the number of RUN302 colonies. On the contrary, when each of the two UW551 mutant strains was co-inoculated with RUN302, only 10 and 19 out of 50 were colonies of the prophage mutant strain and the *orf14* mutant strain, respectively, significantly less than the number of RUN302 colonies in the stems (**Figure 5**).

**FIGURE 6:**
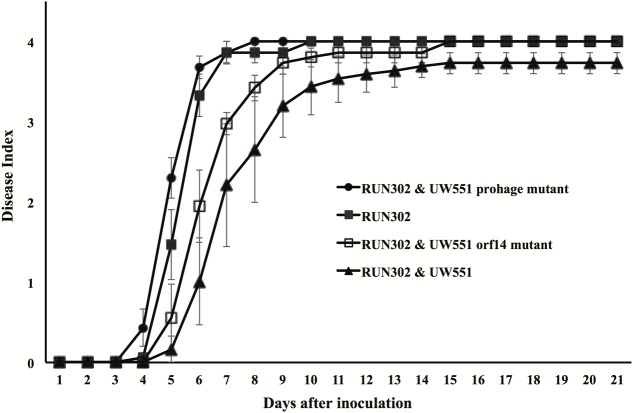
Effect of wt or mutant UW551 strain on the virulence of RUN302 strain of *R. solanacearum*. Two- to three-week-old tomato plants were inoculated by soil drenching with 50 ml (5 × 10^7^ cells/ml) of RUN302 alone, or with a 1:1 mixture of two different *R. solanacearum* strains as indicated. Plants were rated using a disease index of 0 (no wilting) to 4 (76–100% leaves wilted). Points represent means of three separate experiments with 10 plants in each experiment for a total of 30 plants. Bars indicate standard errors.

To study the effect of UW551 and its mutant strains on the virulence of *R. solanacearum* strain RUN302, tomato plants were inoculated by soil drenching with RUN302 alone, or together (1:1) with the wt UW551, the UW551 *orf14* mutant or the UW551 prophage mutant, respectively (**Figure 6**). When RUN302 was co-inoculated with UW551, the co-inoculation caused a delayed and significantly lower DI 4 days after inoculation than inoculation with RUN302 alone (**Figure 6**). On the contrary, when RUN302 was co-inoculated with the UW551 prophage mutant, its overall virulence was similar to that caused by RUN302 alone (**Figure 6**). DIs caused by RUN302 co-inoculated with the UW551 *orf14* mutant strain were lower than the ones caused by RUN302 alone or co-inoculation with RUN302 and the UW551 prophage mutant, but the difference was not significant 9 days after inoculation and all inoculated plants were completely wilted 6 days after that (**Figure 6**).

## Discussion

Currently, a wide range of *R. solanacearum* phages have been reported including filamentous phages of the family *Inoviridae* (Yamada et al., 2007; Murugaiyan et al., 2011; Van et al., 2014), and icosahedral phages of the families *Myoviridae* (Yamada et al., 2007; Bhunchoth et al., 2015), *Podoviridae* (Kawasaki et al., 2009, 2016; Bhunchoth et al., 2015), and *Siphoviridae* (Thi et al., 2015). Comparative genomics also revealed that *R. solanacearum* genomes contain many prophages of the families *Inoviridae* and *Myoviridae* (Yamada et al., 2007; Murugaiyan et al., 2011; Ahmad et al., 2017). The co-evolution between bacteria and bacteriophages plays a key role in driving and maintaining the ecology and evolution of microbial populations, and phages are known to change competitive dynamics among bacterial strains or species (Bohannan and Lenski, 2000a,b; Joo et al., 2006; Koskella et al., 2012; Koskella and Brockhurst, 2014). Temperate phages like the SMP phage of *Streptococcus suis* and the prophages of *Pseudomonas aeruginosa* affect their carrier bacteria in many ways (e.g., growth rate for the former and competitiveness for the latter), contributing to the fitness and virulence of the bacteria (Davies et al., 2016). Previous studies of *R. solanacearum* phages, however, had been focused exclusively on genomic characterization, integration mechanism, effect on their susceptible *R. solanacearum* strains and potential as biocontrols (Kawasaki et al., 2007a,b; Yamada et al., 2007; Askora et al., 2009, 2011; Addy et al., 2012a,b). Our study is the first toward a better understanding of the co-existence between a lysogenic phage and its carrier plant pathogenic bacterial strain by determining the effect of the prophage ϕRs551, through mutagenesis, on the virulence and competitive fitness of its host strain UW551 of *R. solanacearum*.

Recently, we found that the filamentous phage ϕRs551 was stably maintained in the genome of the race 3 biovar 2 strain UW551 of *R. solanacearum* as a prophage, and released to the supernatant of the bacterial strain under normal growth condition (Ahmad et al., 2017). In addition, infection of a ϕRs551-lacking *R. solanacearum* strain RUN302 by the phage caused integration of ϕRs551 into the genome of RUN302, resulting in significantly reduced EPS production, swimming, swarming, and twitching motilities, as well as virulence (Ahmad et al., 2017). In this study, we determined the effect of ϕRs551 on its carrier *R. solanacearum* strain UW551 in virulence and competitive fitness by deleting 3,321 of ϕRs551’s 7,929-bp prophage region from the UW551 genome. As expected, no phage particles were detected, since all but one structural genes of ϕRs551 were deleted in the mutant (**Figure 1**). The deletion also resulted in significantly increased virulence as compared to the wt strain UW551, probably due to the increased EPS production and twitching motility (as indicated by the over-expression of the *pilT* gene), as well as the over-expression of other virulence genes including *egl* and *pehC*, and virulence regulatory genes *phcA* and *hrpB*. Repeated attempts to delete ϕRs551’s entire 7,929-bp prophage region from UW551 were unsuccessful, suggesting that at least a portion of the phage may be essential to *R. solanacearum* UW551 for unknown reasons.

The discovery that the genome of ϕRs551was present with 100% identity in the deposited genomes of 11 race 3 biovar 2 phylotype IIB sequevar 1 strains of *R. solanacearum* isolated from different countries at different times and sequenced independently by different research groups (Ahmad et al., 2017) raised the question about the evolutionary and biological significance of the prophage in the sequevar 1 strains of *R. solanacearum*. Results from our competition assay revealed that ϕRs551 may offer a competitive advantage to its lysogenic host strain UW551 by out-competing its prophage Rs551-lacking strain RUN302 in a mixed infection, since the number of UW551 colonies was significantly higher than that of the RUN302 ones in infected tomato stems only when the wt strain UW551, but not the UW551 prophage mutant, was co-inoculated with RUN302. In virulence, the wt UW551 significantly reduced the virulence of RUN302 when the two strains were co-inoculated, similar to the effect caused by infection of RUN302 by ϕRs551 (Ahmad et al., 2017). The UW551 prophage mutant, however, had little effect on the virulence of RUN302 in the mixed infection of tomato plants, when the prophage of ϕRs551 was mutated (**Figure 6**). The results from our previous and current studies suggest that ecologically, ϕRs551 may offer a competitive advantage to its host *R. solanacearum* by rendering the lysogenic *R. solanacearum* strain less virulent in plant hosts and more dominant over ϕRs551-lacking *R. solanacearum* strains when occupying the same niche, and therefore more persistent in the environment such as plant, soil, and water. This in turn prolongs the symbiotic relationship between ϕRs551 and UW551.

Since ϕRs551 contains a putative type-2 repressor gene *orf14*, it is possible that the observed physiological changes in the UW551 prophage mutant of *R. solanacearum* are due to the lack of transcriptional repression of bacterial virulence-related genes by the phage’s repressor. Such a hypothesis has also been proposed by Addy et al. (2012a) for the type-1 phage repressor. Since the UW551 prophage mutant contains a mutation of *orf14*, we generated the UW551 *orf14* mutant by deleting a 342-bp prophage region from the UW551 genome (which includes the 291-bp *orf14* of ϕRs551) to determine if *orf14* solely played a role in the observed physiological changes in the prophage mutant. In contrast with the UW551 prophage mutant, the UW551 *orf14* mutant produced phage particles like the wt strain UW551. Similar to the prophage mutant strain, however, the *orf14* mutant was significantly increased in production of EPS and expression of the virulence and virulence regulatory genes assayed. This may lead to increased virulence of the *orf14* mutant as compared to the wt strain UW551, although the level of increase was not as high as the prophage mutant strain, suggesting that the observed physiological changes in the prophage mutant are at least partially caused by deletion of the *orf14* gene. The partial effect of the type 2 repressor encoded by *orf14* of ϕRs551 is different from the type 1 repressor encoded by *orf15* of another filamentous *R. solanacearum* phage RSM3, since the loss of virulence caused by infection with ϕRSM3 can be fully restored when the ORF15 of ϕRSM3 was deleted (Addy et al., 2012a). This suggests that the type 2 phage repressor in ϕRs551 may not regulate virulence as tightly as the type 1 repressor in ϕRSM3. Results from our competition assays revealed that like the UW551 prophage mutant, the UW551 *orf14* mutant was significantly out competed by strain RUN302 in colonizing tomato stems (**Figure 5**), and had little effect on the virulence of RUN302 9 days after mixed infection of tomato plants (**Figure 6**). These results suggest that *orf14* may only be partially responsible for offering competitive fitness to the wt strain UW551 in tomato stems and in reducing virulence of the ϕRs551-lacking strain RUN302 in mixed infection. This partial effect of the UW551 *orf14* mutant may be explained by continued production of phage particles in the mutant, thereby exhausting the energy of the mutant or triggering other physiological changes in the mutant and/or host plant.

It is unclear why the expression of the *pilT* gene in the UW551 *orf14* mutant was 21.1-fold higher, but the UW551 prophage mutant was only 8.4-fold higher than the wt strain. Under a microscope, the size of the corrugated trajectories around the colonies of the mutant also looked bigger in the *orf14* mutant than in the prophage mutant (**Figure 2**). Future studies are needed to determine how ORF14 regulates the expression of the *pilT* gene and the significance of PilT in other biological functions other than twitching motility in strain UW551 of *R. solanacearum*.

## Conclusion

We demonstrated that the prophage Rs551 affects multiple important physiological functions of and offers competitive fitness to its carrier *R. solanacearum* strain UW551, at least partially through the type 2 phage repressor encoded by *orf14*. Future research, however, is needed to determine exactly how the phage repressor regulates these functions in the bacterial strain, and what other phage factors contribute to the virulence and competiveness fitness of the carrier bacterial strain against other ϕRs551-lacking *R. solanacearum* strain occupying the same environment and competing for the same ecological niche for plant infection and survival. A better understanding of the relationship between the phage and the bacterium will facilitate effective control of *R. solanacearum*.

## Author Contributions

Conceived and designed the experiments: AA, MS, and QH. Performed the experiments: AA and MS. Analyzed the data: AA and QH. Contributed reagents/materials/analysis tools: QH. Wrote the paper: AA and QH.

## Conflict of Interest Statement

The authors declare that the research was conducted in the absence of any commercial or financial relationships that could be construed as a potential conflict of interest.

## Footnotes

1 http://vassarstats.net/anova1u.html
